# A diagnostic dilemma between epithelioid leiomyoma and PEComa in the right retroperitoneum: A case report of risk-adapted management with long-term recurrence-free follow-up

**DOI:** 10.1097/MD.0000000000048916

**Published:** 2026-05-22

**Authors:** Yangqing Cui, Xuemin Liang, Shiyong Liu, Gangling Su

**Affiliations:** a Guangzhou University of Chinese Medicine, Guangzhou, China; b The Third Affiliated Hospital, Guangzhou University of Chinese Medicine, Guangzhou, China.

**Keywords:** diagnostic dilemma, epithelioid leiomyoma, HMB45, PEComa

## Abstract

**Rationale:**

The accurate classification of retroperitoneal neoplasms remains challenging, particularly when distinguishing between epithelioid leiomyoma and perivascular epitheloid cell tumor (PEComa) due to significant histological and immunohistochemical overlap. This distinction carries profound therapeutic implications, as leiomyomas are benign while PEComas possess malignant potential. The diagnostic dilemma is compounded by the documented phenomenon of aberrant HMB45 expression in otherwise conventional leiomyomas.

**Patient concerns:**

A 47-year-old woman presented with an incidentally discovered right retroperitoneal mass.

**Diagnoses:**

Following complete surgical resection, pathological examination revealed a tumor composed of uniform epithelioid cells with minimal atypia, exceptionally low mitotic activity (1/entire specimen), and low Ki-67 index (2%). Immunohistochemistry showed strong diffuse smooth muscle marker expression (Desmin, h-Caldesmon) but focal HMB45 positivity. Despite this atypical immunophenotype, the diagnosis favored epithelioid leiomyoma based on predominant benign features.

**Interventions:**

A multidisciplinary team recommended against adjuvant therapy, implementing instead a structured surveillance protocol.

**Outcomes:**

The patient remains disease-free after > 4 years of follow-up.

**Lessons:**

This case suggests that, for carefully selected patients with retroperitoneal tumors exhibiting focal HMB45 expression yet predominant benign histological features, a risk-adapted management strategy of complete surgical resection followed by active surveillance may represent a feasible and safe approach. The favorable long-term outcome provides supportive evidence that prioritizing biological behavior over diagnostic nomenclature alone can avoid overtreatment while maintaining oncologic safety. It should be acknowledged that, in the absence of confirmatory molecular studies, the favored diagnosis of epithelioid leiomyoma cannot be considered definitive and PEComa remains a differential consideration. This strategy may serve as a reference for managing similar lesions in the diagnostic gray zone, though larger cohort studies are required to further validate this approach.

## 1. Introduction

The retroperitoneum represents an anatomical compartment characterized by its substantial volume and complex anatomical relationships, creating an environment where a diverse spectrum of both primary and secondary neoplasms can develop. Epidemiological studies consistently demonstrate that the majority of primary retroperitoneal masses are malignant, with soft tissue sarcomas accounting for a significant proportion of these cases.^[1]^ The diagnostic challenge inherent to this anatomical site is compounded by its spacious nature, which permits tumors to attain considerable dimensions before manifesting clinical symptoms, frequently resulting in delayed diagnosis and complex therapeutic interventions.^[2]^ The management of retroperitoneal sarcoma centers on complete en bloc resection performed by specialized surgical oncologists within a multidisciplinary team. While this approach aims to maximize disease clearance, high recurrence rates persist, with patterns dictated by histologic subtype. The roles of radiation and systemic therapy in primary disease are evolving, underscoring the need for international collaboration to define unresectability criteria and optimize treatment strategies.^[3]^ This diagnostic ambiguity underscores the critical importance of precise histopathological characterization, as the accurate classification of these neoplasms directly dictates subsequent management strategies, ranging from conservative surveillance to multimodal therapy, and fundamentally influences long-term patient outcomes.

Within this complex diagnostic landscape, the histopathological distinction between epithelioid leiomyoma, a rare benign neoplasm of smooth muscle origin, and PEComa, a mesenchymal tumor of unpredictable biological behavior, represents a significant diagnostic challenge for both pathologists and clinicians.^[4]^ This diagnostic dilemma carries profound clinical implications, as their management pathways diverge substantially. Epithelioid leiomyomas are universally benign and typically cured by complete surgical excision alone. In contrast, PEComas demonstrate a spectrum of biological potential; according to established risk stratification criteria, malignant variants may necessitate extended resection margins, adjuvant therapy, and rigorous long-term surveillance due to their documented potential for local recurrence and distant metastasis.^[5,6]^

The diagnostic complexity in distinguishing epithelioid leiomyoma from PEComa stems from significant histological and immunophenotypic overlaps. Histologically, both neoplasms are composed of epithelioid cells with abundant clear to eosinophilic cytoplasm, frequently arranged in nested or sheet-like architectures, making purely morphological distinction challenging.^[7,8]^ Immunohistochemically, while the co-expression of smooth muscle markers (e.g., smooth muscle actin (SMA), h-caldesmon) and melanocytic markers (e.g., HMB45, Melan-A) has been established as a diagnostic hallmark of PEComa, this criterion is not pathognomonic.^[9–12]^ Critically, numerous studies have documented that a subset of histologically conventional and epithelioid leiomyomas can demonstrate aberrant, typically focal, expression of HMB45, representing a well-recognized diagnostic pitfall that substantially blurs the diagnostic boundary between these entities.^[13,14]^ This immunophenotypic ambiguity creates a diagnostic “gray zone,” wherein reliance on any single immunohistochemical marker is insufficient and potentially misleading. This challenge of overlapping immunoprofiles is not the sole source of diagnostic difficulty in the retroperitoneum. The spectrum of lesions to be considered also includes entities whose recognition relies on identifying distinct architectural features rather than a single immunomarker. Mucinous cystic neoplasms, for instance, though primarily pancreatic and with a strong female predominance, can occur in the retroperitoneum and have rarely been documented in males, where their defining “ovarian-type” stroma can lead to initial misdiagnosis when the clinical context is atypical.^[15]^ Consequently, an accurate diagnosis necessitates an integrative approach that critically prioritizes key morphological features, including mitotic rate, tumor necrosis, and nuclear atypia, within the context of the complete clinical presentation and immunohistochemical profile.^[16]^ This comprehensive diagnostic strategy is essential for avoiding misclassification and ensuring appropriate clinical management.

Herein, we report a diagnostically challenging case of a right retroperitoneal epithelioid leiomyoma in a 47-year-old woman, initially suspected to be an angiomyolipoma on preoperative imaging. The pathological diagnosis was complicated by focal HMB45 immunoreactivity, which introduced PEComa as a significant differential consideration. This case offers 2 instructive observations for the management of such diagnostically ambiguous retroperitoneal tumors. First, it exemplifies a rational, evidence-based clinical decision-making process in which a risk-adapted management strategy, omitting adjuvant therapy in favor of structured surveillance, was implemented based on predominant benign histological characteristics, notwithstanding the diagnostic uncertainty. Second, the long-term follow-up of over 4 years without evidence of recurrence provides supportive clinical evidence for the safety and efficacy of this tailored therapeutic approach. It should be noted that, in the absence of confirmatory molecular testing, epithelioid leiomyoma represents a favored diagnosis rather than a definitive one. By presenting this case, we aim to contribute to the clinical discourse on managing retroperitoneal tumors exhibiting overlapping pathological features.

## 2. Case presentation

A 47-year-old married woman was admitted to our urology department for surgical evaluation of a right retroperitoneal mass that had been incidentally discovered 2 years prior. The mass was initially detected during a routine health examination ultrasound, which was interpreted as suggesting a possible duplicated renal system (based on patient recall; the original report was unavailable). Throughout this 2-year period, the patient remained entirely asymptomatic, reporting no back pain, abdominal discomfort, urinary abnormalities, gross hematuria, headaches, dizziness, palpitations, chest tightness, abnormal sweating, significant weight loss, fever, or bone pain. Consequently, she sought no further medical intervention.

Her medical history was significant for uterine leiomyomas, for which she had undergone multiple laparoscopic myomectomies and a bilateral tubal ligation. Her obstetric history was gravida 5, with 4 therapeutic abortions and one full-term vaginal delivery. Her spouse and children were healthy, and she had no future fertility desires. Systemic review was unremarkable for chronic conditions (e.g., hypertension, coronary heart disease, diabetes), infectious diseases (e.g., hepatitis, typhoid, tuberculosis), prior trauma, other surgeries, blood transfusions, or drug and food allergies. There was no family history of malignancy. Her vaccination history was unclear.

**Figure 1.**
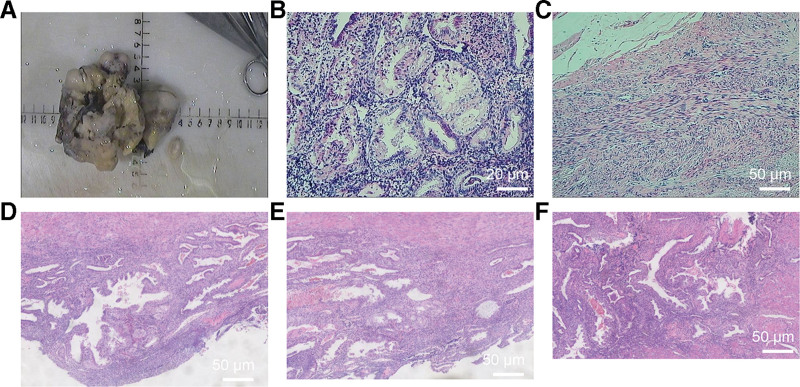
Gross and histopathological examination of the hysterectomy with bilateral salpingectomy specimen.

**Figure 2.**
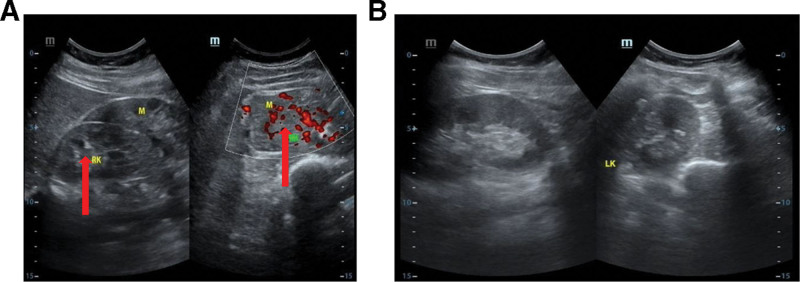
Preoperative ultrasonographic characterization of a large right retroperitoneal mass.

**Figure 3.**
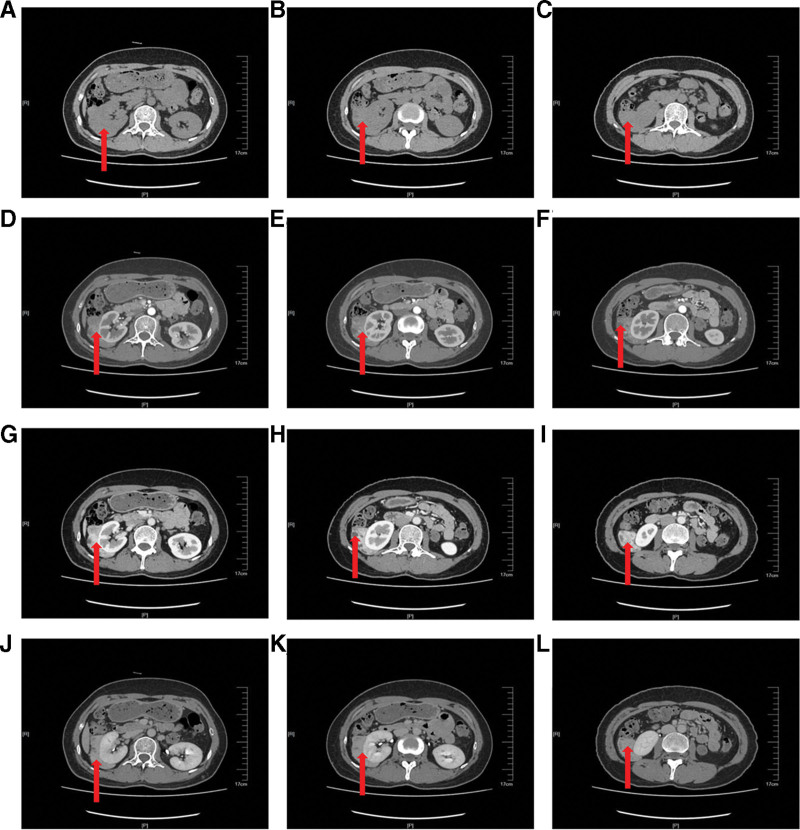
Preoperative renal CT reveals a large extrarenal mass, suggestive of a fat-poor AML. AML = angiomyolipoma, CT = computed tomography.

The patient was subsequently admitted for a total hysterectomy and bilateral salpingectomy due to multiple recurrent uterine leiomyomas. Pathological examination of the hysterectomy specimen (Fig. 1) revealed endometrial simple hyperplasia with focal complex atypical hyperplasia, multiple uterine leiomyomas, adenomyosis, chronic cervicitis and endocervicitis, and chronic salpingitis bilaterally, with unremarkable parametrial tissues. Following the detection of an indeterminate right perirenal mass on abdominal ultrasound (Fig. 2), a preoperative renal computed tomography (CT) scan (Fig. 3) was obtained. It characterized the lesion as a solid, fat-poor extrarenal mass, highly suggestive of an angiomyolipoma, thereby providing the rationale for surgical intervention.

Following urological consultation, surgical resection of the retroperitoneal mass was recommended. After an uneventful recovery from her gynecological surgery, the patient was readmitted to the urology department. She subsequently underwent resection of the right retroperitoneal mass under general anesthesia. The procedure began laparoscopically but was converted to an open approach due to profuse hemorrhage from the highly vascularized tumor surface that was refractory to bipolar coagulation. Intraoperative findings confirmed the tumor was located on the anterolateral aspect of the upper pole of the right kidney, densely adherent to the renal parenchyma over an area of approximately 1 cm, with fused capsules. The tumor was completely resected. Active bleeding from the renal surface was successfully controlled with compression and 3 absorbable sutures.

**Figure 4.**
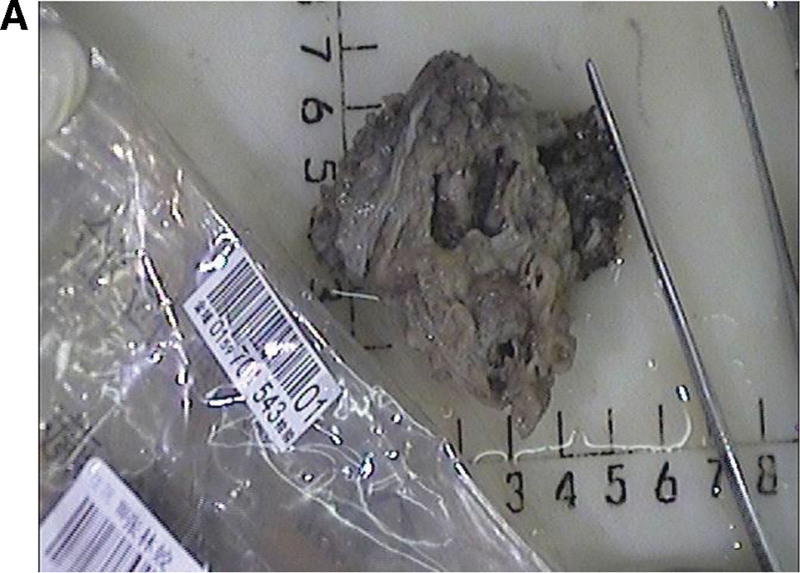
Gross appearance of the resected retroperitoneal tumor.

**Figure 5.**
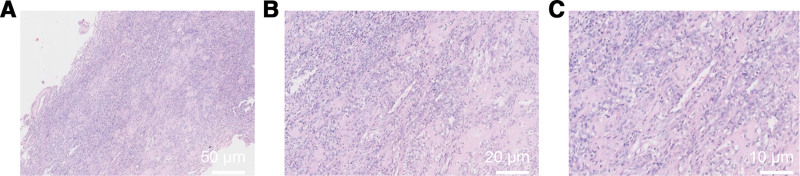
H&E-stained section guiding diagnostic workflow.

**Figure 6.**
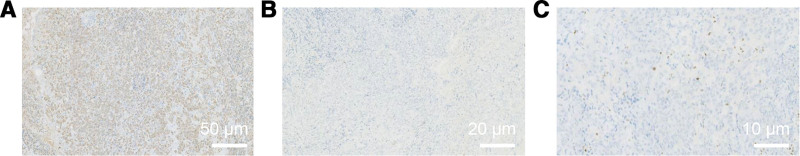
Immunohistochemical profiling of the retroperitoneal tumor.

Pathological Findings: Gross examination of the resected retroperitoneal mass revealed multiple cystic-wall-like tissue fragments aggregating to 6 cm × 6 cm × 2 cm, with a wall thickness of 0.1 cm–0.3 cm (Fig. 4). On H&E-stained sections (Fig. 5), the tumor displayed solid, sheet-like growth of monotonous cells with pale cytoplasm and infrequent mitoses, supported by a rich vascular stroma. To further characterize this neoplasm and establish its lineage, immunohistochemical studies were performed. Immunohistochemical (Fig. 6) profiling yielded the following results:

- Smooth muscle markers: Desmin (diffusely positive), SMA (focally positive), h-Caldesmon (partially positive), Calponin (positive).
- Melanocytic markers: HMB45 (positive in rare, scattered individual cells), Melan-A (negative).
- Vascular marker: CD34 (positive in vascular endothelium).
- Other markers: Vimentin (positive), CD99 (positive), BCL2 (positive), WT-1 (partially positive).
- Negative markers: CK, S100, D2-40, STAT6, epithelial membrane antigen, Myogenin, MyoD1.
- Proliferation index: Ki-67 was low at approximately 2%.

Diagnosis, management, and follow-up: The pathological features were most consistent with a benign epithelioid leiomyoma, albeit with atypical immunohistochemical features. This interpretation was primarily based on the tumor’s solid architecture of uniform Desmin-positive cells, very low proliferative activity (1 mitotic figure, low Ki-67 index), and the patient’s pertinent history of uterine leiomyomas. The diagnosis was complicated by focal HMB45 expression, a feature shared by PEComa. The considerable immunophenotypic overlap between these entities precluded a definitive diagnosis based on pathology alone, highlighting a diagnostic gray zone in soft tissue pathology, necessitating final interpretation within the full clinical context.

A multidisciplinary team discussion was held. Considering the overwhelming evidence of benign biological behavior (very low mitotic and proliferative activity) achieved with complete surgical excision, a conservative management strategy was adopted. No adjuvant radiotherapy or chemotherapy was administered. A stringent active surveillance protocol was implemented, involving renal ultrasonography every 6 months for the first 2 years, then annually, with planned contrast-enhanced CT scans every 2-3 years for at least 5 years.

**Figure 7.**
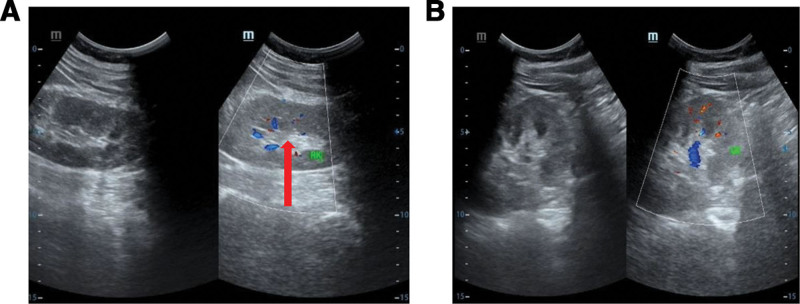
Surveillance ultrasound within 2 months postoperatively following complete resection of a right-sided retroperitoneal tumor.

**Figure 8.**
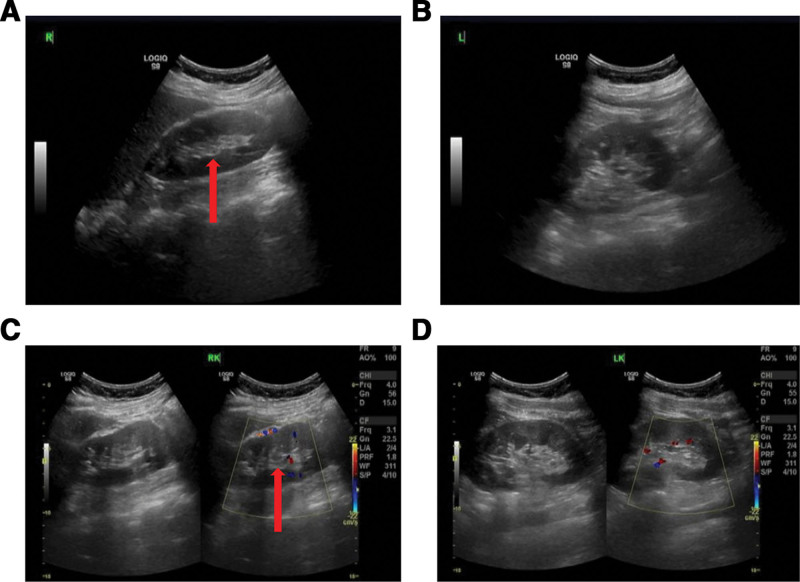
Serial ultrasonographic surveillance demonstrates absence of local recurrence in the first postoperative year after complete resection of a right-sided retroperitoneal tumor.

**Figure 9.**
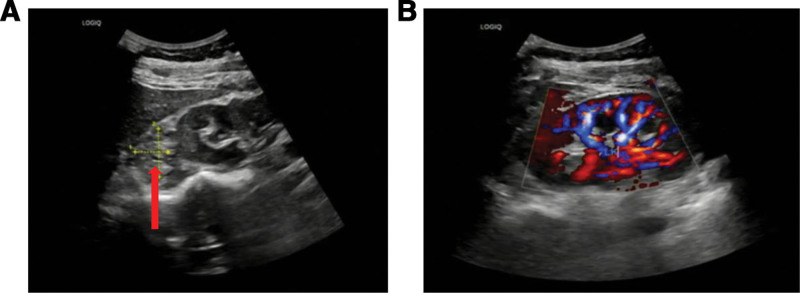
Equivocal ultrasound finding at 4-year follow-up.

**Figure 10.**
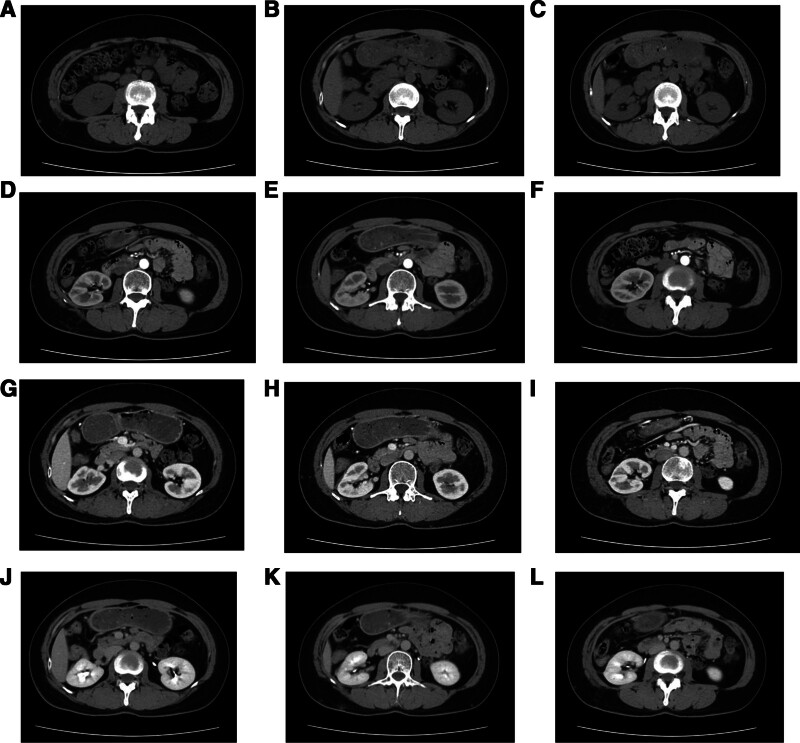
Four-year postoperative surveillance with non-contrast and contrast-enhanced renal CT. CT = computed tomography.

The patient has been maintained on a structured surveillance protocol for over 4 years. Follow-up renal ultrasonography (Fig. 7) performed at 2 months postoperatively demonstrated normal findings. During the first postoperative year, 2 additional ultrasound examinations (Fig. 8) were conducted, both confirming the absence of recurrence showed normal findings. During the postoperative fourth-year follow-up, surveillance ultrasonography identified an equivocal finding (Fig. 9), leading to subsequent contrast-enhanced CT evaluation (Fig. 10). Careful assessment of both imaging modalities confirmed absence of tumor recurrence. The patient’s maintained disease-free status further validates the original diagnostic rationale and conservative treatment approach.

## 3. Discussion

Diagnostic characterization of retroperitoneal neoplasms continues to pose substantial challenges in surgical pathology practice, particularly when evaluating lesions that demonstrate overlapping histomorphological features and ambiguous immunophenotypic profiles. This diagnostic complexity is particularly pronounced in the distinction between rare benign entities and tumors with uncertain malignant potential, where immunohistochemical markers traditionally considered specific may exhibit unexpected cross-reactivity.^[17]^ The present case of a right retroperitoneal epithelioid leiomyoma with aberrant HMB45 expression aptly illustrates this diagnostic conundrum. This case not only highlights the critical limitations of relying on single immunohistochemical markers in isolation but also provides a compelling clinical rationale for implementing a comprehensive, multidisciplinary approach to diagnosis and management in such diagnostically ambiguous scenarios. Furthermore, it underscores the necessity of integrating complete histological features with clinical presentation and follow-up data to achieve optimal patient outcomes, thereby serving as an instructive example of evidence-based decision-making in challenging diagnostic contexts.

The favored pathological diagnosis in this case was established through a comprehensive evaluation of multiple concordant features, collectively providing compelling evidence for epithelioid leiomyoma as the most likely interpretation. Histological assessment revealed a tumor characterized by an entirely benign profile, exhibiting minimal cytologic atypia, exceptionally low mitotic activity (with only a single mitotic figure identified throughout all sampled sections), and a low proliferative index as demonstrated by Ki-67 immunostaining (approximately 2%). Immunohistochemically, the neoplasm showed strong and diffuse expression of well-established smooth muscle markers, including Desmin and h-Caldesmon, confirming smooth muscle differentiation. While the observed focal HMB45 immunoreactivity initially suggested PEComa in the differential diagnosis, this finding requires careful contextual interpretation within the complete diagnostic spectrum. Of critical diagnostic importance is the recognition that focal HMB45 immunoreactivity represents a well-characterized immunophenotypic variant within the pathological spectrum of smooth muscle neoplasms. Substantial clinicopathological evidence from multiple independent studies has systematically established that this immunoprofile occurs reproducibly in histologically conventional leiomyomas.^[13,14]^ Consequently, this finding should be interpreted as a potential diagnostic mimic rather than pathognomonic evidence of PEComa differentiation. These investigations consistently demonstrate that limited melanocytic marker expression in a distinct subset of leiomyomas does not confer the aggressive biological behavior characteristic of true PEComas.^[9–12]^ This diagnostic nuance necessitates a comprehensive interpretive framework wherein such aberrant immunoreactivity should be weighed against the dominant morphological and immunohistochemical profile rather than being accorded disproportionate diagnostic significance. In such circumstances, isolated HMB45 positivity should not supersede an otherwise convincing diagnosis of smooth muscle neoplasia. The concurrent negativity for Melan-A, another melanocytic marker typically expressed in PEComas, further diminishes the diagnostic weight of the focal HMB45 reactivity, reinforcing its interpretation as a potential diagnostic distraction rather than a definitive indicator of PEComa differentiation.^[17,18]^ Nevertheless, in the absence of confirmatory molecular studies such as TFE3 rearrangement analysis or TSC1/TSC2 mutation testing, which were not available in this case, PEComa cannot be categorically excluded and should remain a differential consideration.^[19–21]^ Accordingly, we present epithelioid leiomyoma as the favored diagnosis based on the preponderance of pathological evidence.

The management of this case highlights the critical importance of risk-adapted clinical decision-making grounded in meticulous pathological assessment. The consensus to pursue a risk-adapted course, complete surgical resection without adjuvant therapy, was predicated on a thorough evaluation of the tumor’s biological behavior rather than diagnostic nomenclature alone. Established risk-stratification models for PEComa definitively associate aggressive clinical behavior with specific histological features, including elevated mitotic activity, tumor necrosis, and infiltrative growth patterns.^[22]^ Our case demonstrated a complete absence of these high-risk determinants, instead exhibiting uniformly indolent features such as an exceptionally low mitotic count and low proliferative index. In this context, administering radiotherapy or systemic chemotherapy would have exposed the patient to significant treatment-related toxicity without established clinical benefit. This case thus exemplifies the fundamental principle of tailoring treatment intensity to the individual risk profile of the tumor, a cornerstone of sound surgical oncology that prioritizes the avoidance of overtreatment for biologically indolent neoplasms.

The long-term follow-up data, demonstrating sustained disease-free status over a period exceeding 4 years, provides supportive clinical evidence for our diagnostic reasoning and therapeutic strategy. This favorable oncological outcome suggests that for retroperitoneal neoplasms exhibiting predominantly low-risk pathological features, a management approach centered on complete surgical excision followed by structured active surveillance may represent a feasible and safe approach in carefully selected cases. Furthermore, this extended period of tumor quiescence significantly reinforces the diagnostic likelihood of epithelioid leiomyoma as the favored interpretation in this context, supporting the view that such a diagnosis can be reasonably maintained when supported by convergent clinical and pathological evidence, even when confronted with equivocal immunohistochemical findings that might otherwise suggest alternative diagnoses. This case thus suggests a clinically instructive approach, wherein the predictive value of a cohesive clinicopathological profile can outweigh the diagnostic uncertainty introduced by isolated aberrant immunohistochemical markers in guiding both prognosis and optimal management strategies for retroperitoneal tumors.

While this case provides valuable clinical insights, several methodological limitations merit consideration. Primarily, the diagnostic evaluation was constrained to conventional histopathological and immunohistochemical methodologies. Advanced molecular analyses, including TFE3 fluorescence in situ hybridization to exclude TFE3-rearranged PEComa variants or next-generation sequencing for tuberous sclerosis complex (TSC)1/TSC2 mutations that characterize a subset of PEComas, were not performed.^[19–21]^ Although such specialized testing could have provided additional diagnostic certainty, it is important to note that these modalities have not yet been established as routine requirements for diagnosing epithelioid leiomyoma or PEComa in standard clinical practice. Secondly, the surgical margins were not subjected to dedicated pathological assessment, as routine pathologic protocols primarily focused on lesional characterization rather than margin evaluation. Nevertheless, we recognize that in tumors with borderline malignancy potential, margin status holds both diagnostic and therapeutic relevance. Histologically positive margins would have suggested a more aggressive course and potentially indicated re-excision,^[23]^ while confirmed negative margins would have provided additional supportive evidence for the benign diagnosis and corroborated the conservative management approach implemented in this case. Finally, as with all single-case reports, the generalizability of our findings remains inherently limited. The conclusions drawn from this individual experience require validation through larger, multi-institutional cohort studies that would enable more robust statistical analysis and enhance the external validity of our proposed management framework. Consequently, the diagnosis of epithelioid leiomyoma, while strongly favored by histomorphological and immunophenotypic features, is best regarded as a clinicopathologically reasoned conclusion rather than a molecularly confirmed definitive diagnosis.

This meticulously documented case illuminates a well-recognized yet complex diagnostic dilemma in soft tissue pathology, the distinction between epithelioid leiomyoma and PEComa in the retroperitoneum. It suggests that through rigorous histopathological evaluation and thoughtful immunohistochemical interpretation, diagnostically challenging cases can often be successfully navigated using existing clinical tools. Most significantly, this report provides supportive evidence for a risk-adapted management approach when pathological assessment reveals predominantly indolent features, even in the presence of ambiguous immunohistochemical findings. The favorable long-term outcome achieved through complete surgical excision followed by structured surveillance provides preliminary support for the feasibility and safety of this approach in appropriately selected patients. This case further reinforces the importance of a multidisciplinary team approach, whereby clinical, radiological, and pathological data are collectively reviewed to reach a consensus on diagnosis and management, ensuring that treatment intensity is appropriately calibrated to the tumor’s biological behavior. Ultimately, this case adds to the growing body of clinical evidence on retroperitoneal tumor management and provides a reference for addressing similar diagnostically ambiguous scenarios in clinical practice. It supports the fundamental principle that therapeutic decisions should be guided by comprehensive assessment of a tumor’s biological behavior rather than overreliance on potentially misleading individual diagnostic elements. It should be acknowledged, however, that in the absence of molecular confirmation, the diagnosis of epithelioid leiomyoma remains a favored interpretation and PEComa cannot be entirely excluded. Definitive conclusions await validation through larger multi-institutional studies.

## 4. Conclusions

This case supports the diagnostic principle that in retroperitoneal tumors with ambiguous immunophenotypes, the preponderance of morphological evidence for biological indolence should guide clinical decision-making, even when isolated aberrant immunohistochemical findings introduce diagnostic uncertainty. Our experience, with a favorable outcome over more than 4 years of follow-up, suggests that a risk-adapted management strategy, consisting of complete surgical resection followed by structured active surveillance, may represent a feasible and safe approach for carefully selected patients presenting with lesions in the diagnostic gray zone between epithelioid leiomyoma and PEComa. This risk-adapted paradigm, which tailors treatment intensity to the tumor’s histological risk profile rather than diagnostic nomenclature alone, has the potential to avoid the morbidity of overtreatment while achieving satisfactory oncologic outcomes. However, it must be acknowledged that, in the absence of confirmatory molecular studies such as TFE3 rearrangement analysis or TSC1/TSC2 mutation testing, the diagnosis of epithelioid leiomyoma remains a favored interpretation rather than a definitive one, and PEComa cannot be categorically excluded. Therefore, this case should be viewed as providing supportive clinical evidence for the feasibility of a risk-adapted approach in appropriately selected low-risk cases, rather than as definitive validation of a broadly applicable management paradigm. Larger multi-institutional cohort studies with extended follow-up are warranted to further substantiate the safety and efficacy of this strategy.

## Author contributions

**Data curation:** Yangqing Cui, Gangling Su.

**Writing – original draft:** Yangqing Cui, Gangling Su.

**Writing – review & editing:** Yangqing Cui, Xuemin Liang, Shiyong Liu, Gangling Su.
